# Predicting Spike Features of Hodgkin-Huxley-Type Neurons With Simple Artificial Neural Network

**DOI:** 10.3389/fncom.2021.800875

**Published:** 2022-02-07

**Authors:** Tian Wang, Ye Wang, Jiamin Shen, Lei Wang, Lihong Cao

**Affiliations:** ^1^State Key Laboratory of Media Convergence and Communication, Communication University of China, Beijing, China; ^2^Neuroscience and Intelligent Media Institute, Communication University of China, Beijing, China; ^3^State Key Laboratory of Mathematical Engineering and Advanced Computing, Wuxi, China

**Keywords:** spike, Hodgkin-Huxley model, spike features prediction, artificial neural network, spike prediction module, feature prediction module

## Abstract

Hodgkin-Huxley (HH)-type model is the most famous computational model for simulating neural activity. It shows the highest accuracy in capturing neuronal spikes, and its model parameters have definite physiological meanings. However, HH-type models are computationally expensive. To address this problem, a previous study proposed a spike prediction module (SPM) to predict whether a spike will take place 1 ms later based on three voltage values with intervals of 1 ms. Although SPM does well, it fails to evaluate the informative features of the spike. In this study, the feature prediction module (FPM) based on simple artificial neural network (ANN) was proposed to predict spike features including maximum voltage, minimum voltage, and dropping interval. Nine different HH-type models were adopted whose firing patterns cover most of the firing behaviors observed in the brain. Voltage and spike feature samples under constant external input current were collected for training and testing. Experiment results illustrated that the combination of SPM and FPM can accurately predict the spiking part of different HH-type models and can generalize to unseen types of input current. The combination of SPM and FPM may offer a possible way to simulate the action potentials of biological neurons with high accuracy and efficiency.

## 1. Introduction

Neurons communicate with each other in the form of spikes. Spikes of neurons are of great importance in the brain as they contribute to efficient neural information processing and facilitate the transmission of information (Bean, 2007). The generation of spikes depends in large part on voltage-gated ion channels (Berger and Crook, 2015). Neurons embedded with voltage-gated ion channels can almost always generate spikes. Under different densities or combinations of ion channels, they can perform a diversity of firing behaviors, e.g., spiking, bursting, subthreshold oscillation, and mixed-mode (Goldman et al., 2001).

For decades, many computational models have been proposed to simulate the firing behaviors of neurons. These models can be mainly classified into two categories: 1) simplified model without ion channels, and 2) detailed model with ion channels. Representative models in the first category include integrate-and-fire (IF) model (Lapique, 1907; Abbott, 1999), leaky IF model (Stein, 1967), adaptive IF model (Brette and Gerstner, 2005), resonate-and-fire (RF) model (Izhikevich, 2001), FitzHugh-Nagumo (FHN) model (FitzHugh, 1961; Nagumo et al., 1962), Hindmarsh-Rose (HR) model (Hindmarsh and Rose, 1984), Izhikevich model (Izhikevich, 2003), and map-based (MB) model (Rulkov, 2002). While representative models in the second category include Hodgkin-Huxley (HH) model (Hodgkin and Huxley, 1952) and Morris-Lecar (ML) model (Morris and Lecar, 1981).

Simplified models are convenient for numerical calculations and have been widely used in simulating large-scale brain networks or neural circuits. However, as ion channels are the building blocks of spike generation, given their lack of ion channels, simplified models might be inaccurate when capturing spike features of neurons; moreover, parameters in these models have little electrophysiological meanings. Therefore, parameter tunings are time-consuming.

On the contrary, detailed models are much more accurate in characterizing spike features of neurons. The ML model is a special case of the HH model, which aims at simulating the oscillatory behavior of barnacle muscle fiber (Morris and Lecar, 1981). In the remaining part of this paper, detailed models refer to the HH-type models. HH-type models embedded with different ion channels can generate various firing behaviors, reproducing spikes similar to experimental data, and parameters included in these models have definite physiological meanings. However, because of their inconvenience for numerical calculations, HH-type models are difficult to carry out large-scale brain network simulations.

**Figure 1:**
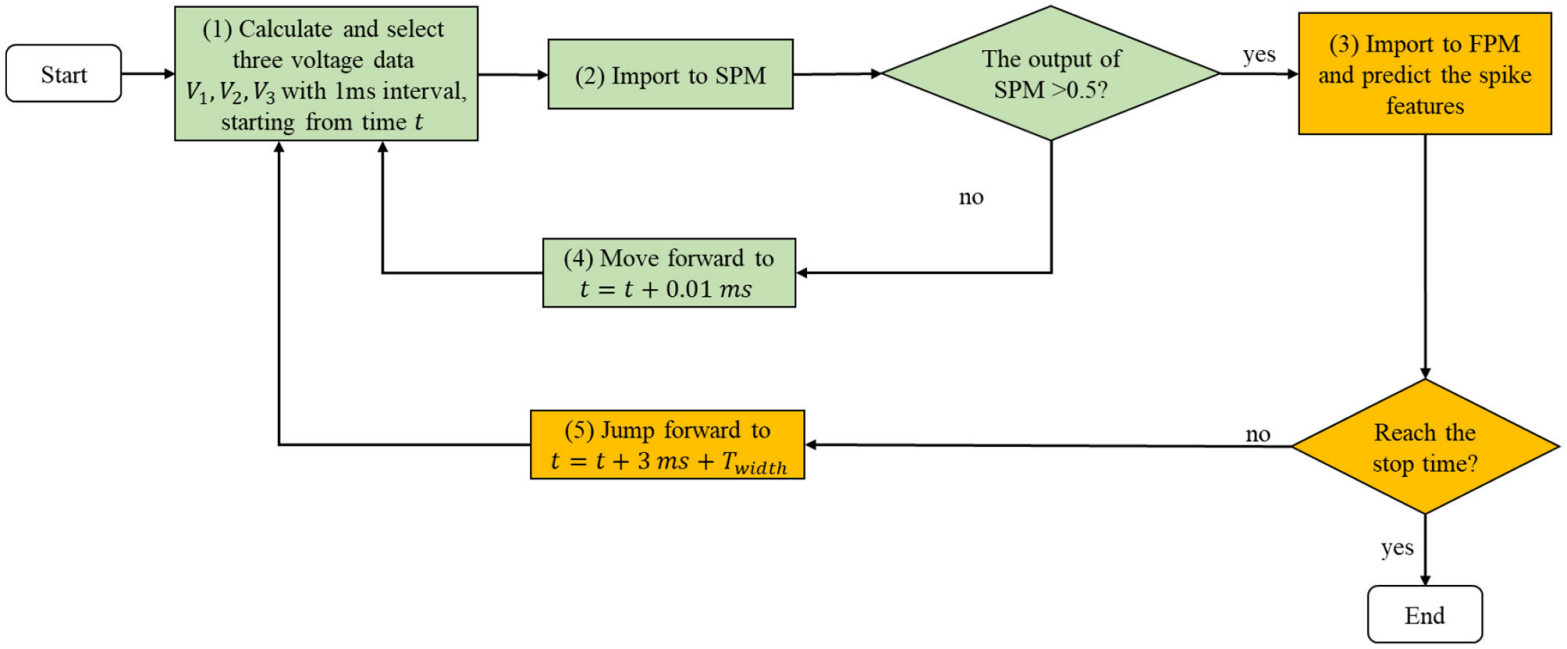
The schematic shows all the processes in the method. Collected sequential *V*_1_, *V*_2_, and *V*_3_ were first imported to the spike prediction module (SPM) to judge whether these three values can be used to predict spike features; if the output of SPM is higher than 0.5 (threshold), then *V*_1_, *V*_2_, and *V*_3_ were imported to the feature prediction module (FPM) to do feature predictions. After that, new *V*_1_, *V*_2_, and *V*_3_ were collected start from *t* + 3*ms* + *T*_*width*_ until the stop time is reached; while if the output of SPM is lower than 0.5, *V*_1_, *V*_2_, and *V*_3_ were moved forward with 0.01 ms to get new *V*_1_, *V*_2_, and *V*_3_ and imported to the SPM to do classifications. These procedures were repeated until reaching the stop time.

Previous studies have tried to accelerate the calculation of the HH-type models in several different ways. For example, researchers increased the time step in numerical schemes, e.g., from 0.01 to 0.1 ms. However, this is not an optimal approach as it may lead to lower simulation accuracy. Recently, another method has been proposed: library-based numerical method Sun et al. (2009). Specifically, plenty of spike samples from the classical HH model under different stimulus intensities were collected to build a library (spike database). When performing network simulation, spike sequences of the corresponding HH neurons can be estimated from the library. However, this method is still not good enough as it can only reproduce raw statistical information of spikes, e.g., average firing rate, interspike interval distribution, and power spectra of voltage traces, and cannot capture the spike timing information of neurons, which is more informative. Moreover, as they only tested their method using the classical HH model, the generalization ability of the method is still unknown. Ionic neuron models have many different types besides classical HH-type and firing activities produced by these neurons are rather diverse and changeable. Previously, Cao et al. proposed a spike prediction module (SPM) to use three voltage values (intervals of 1 ms) to decide whether or not a spike will take place 1 ms later (Cao et al., 2018). Although SPM does well, the features of the spike have not been evaluated. In this study, a novel method named feature prediction module (FPM) was proposed to predict informative spike features including maximum voltage, minimum voltage, and dropping interval. To the best of our knowledge, we are the first to explore the task of spike feature prediction with a simple artificial neural network. Spike features and voltage data were collected from nine different HH-type models whose firing patterns cover most of the firing behaviors observed in the brain. Then, we constructed the FPM based on the SPM. For three sequential voltages (interval of 1 ms), the SPM was first used for spike predictions; if a spike is predicted, then the voltages will be imported to the FPM to predict spike features; otherwise, the voltages will be updated and imported to the SPM again (as shown in Figure 1 for more details).

## 2. Materials and Methods

### 2.1. Overview of the Method

The schematic of all the processes in the method is illustrated in Figure 1.

**Figure 2:**
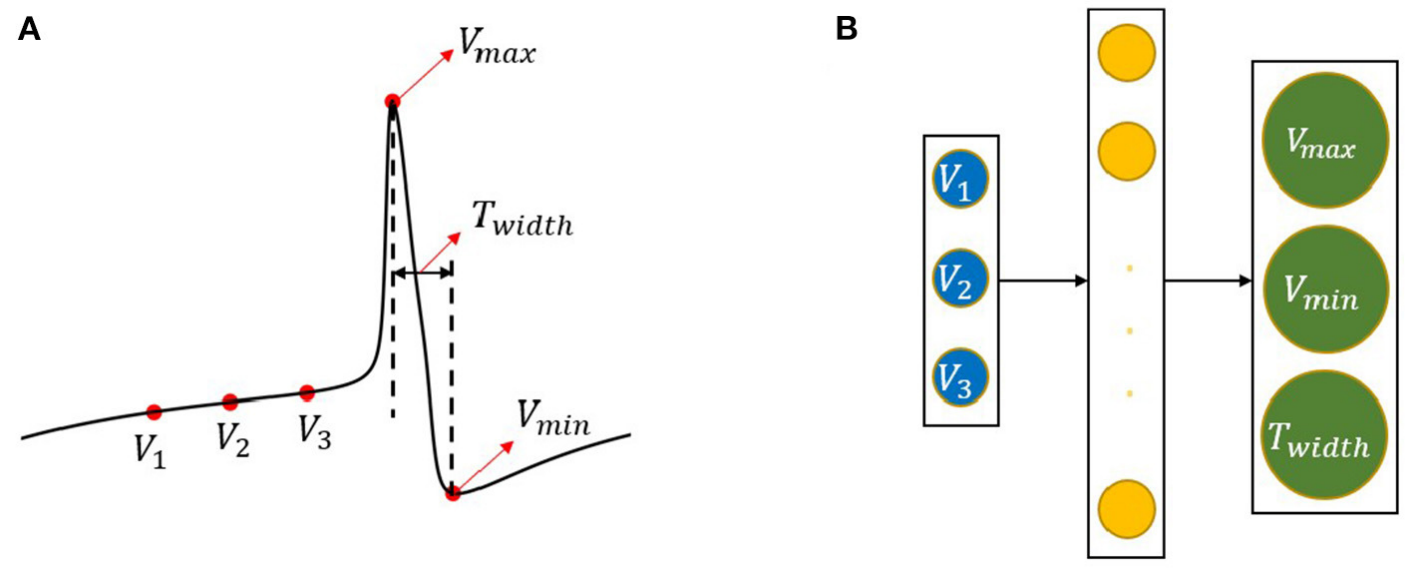
Three typical features of a spike and the FPM structure. **(A)** The *V*_*max*_, *V*_*min*_, and *T*_*width*_ denote the maximum voltage, minimum voltage, and dropping interval of the spike (the difference between *t*_*max*_ and *t*_*min*_), respectively, *V*_1_, *V*_2_, and *V*_3_ denote three sequential voltage values with an interval of 1 ms before *V*_*max*_. **(B)** The structure of FPM, with *V*_1_, *V*_2_, and *V*_3_ as input and *V*_*max*_, *V*_*min*_, and *T*_*width*_ as output. One hidden layer is used with ten artificial neurons.

(1) Three sequential voltage data with 1 ms interval were extracted from a given neuron model.
(2) The SPM was used to judge whether there would be a spike of 1 ms later (Cao et al., 2018).
(3) If the output of SPM is bigger than 0.5, then the input voltages would be imported to the FPM, where we introduced a three-layer artificial neural network (ANN) to do predictions. Specifically, three units were used in the input layer, corresponds to the three input voltage values; ten units were used in the hidden layer to extract features from the input layer; three units were used in the output layer, which corresponds to three spike features (see *V*_*max*_, *V*_*min*_, and *T*_*width*_ in Figure 2).
(4) While if the output of SPM is smaller than 0.5, then the input voltages would move forward with 0.01 ms to acquire new voltage samples and import them to the SPM again.
(5) Following step (3), we predict the spike features and calculate the loss (mean squared error, MSE). After that, we observed whether the stop time is reached; if yes, the whole process would stop; otherwise, voltage samples would be extracted from a new start time: *t*+3*ms*+*T*_*width*_ (*t* denotes time corresponds to *V*_1_), and does classifications and predictions again until the stop time is reached.

### 2.2. Three Typical Features in Neuronal Spikes

Spikes are the basic and crucial units in reflecting neuronal activities. Figure 2A demonstrates a typical spike, marked with three indices, *V*_*max*_, *V*_*min*_, and *T*_*width*_, which denote the maximum voltage, minimum voltage, and dropping interval (time interval between *V*_*max*_ and *V*_*min*_) of the spike, respectively. *V*_1_, *V*_2_, and *V*_3_ are three sequential voltage values before *V*_*max*_, with an interval of 1 ms. *V*_1_, *V*_2_, and *V*_3_ were then imported to FPM, with *V*_*max*_, *V*_*min*_, and *T*_*width*_ as output, ten artificial neurons were used in the hidden layer of FPM, as shown in Figure 2B.

### 2.3. Neuron Models

**Figure 3:**
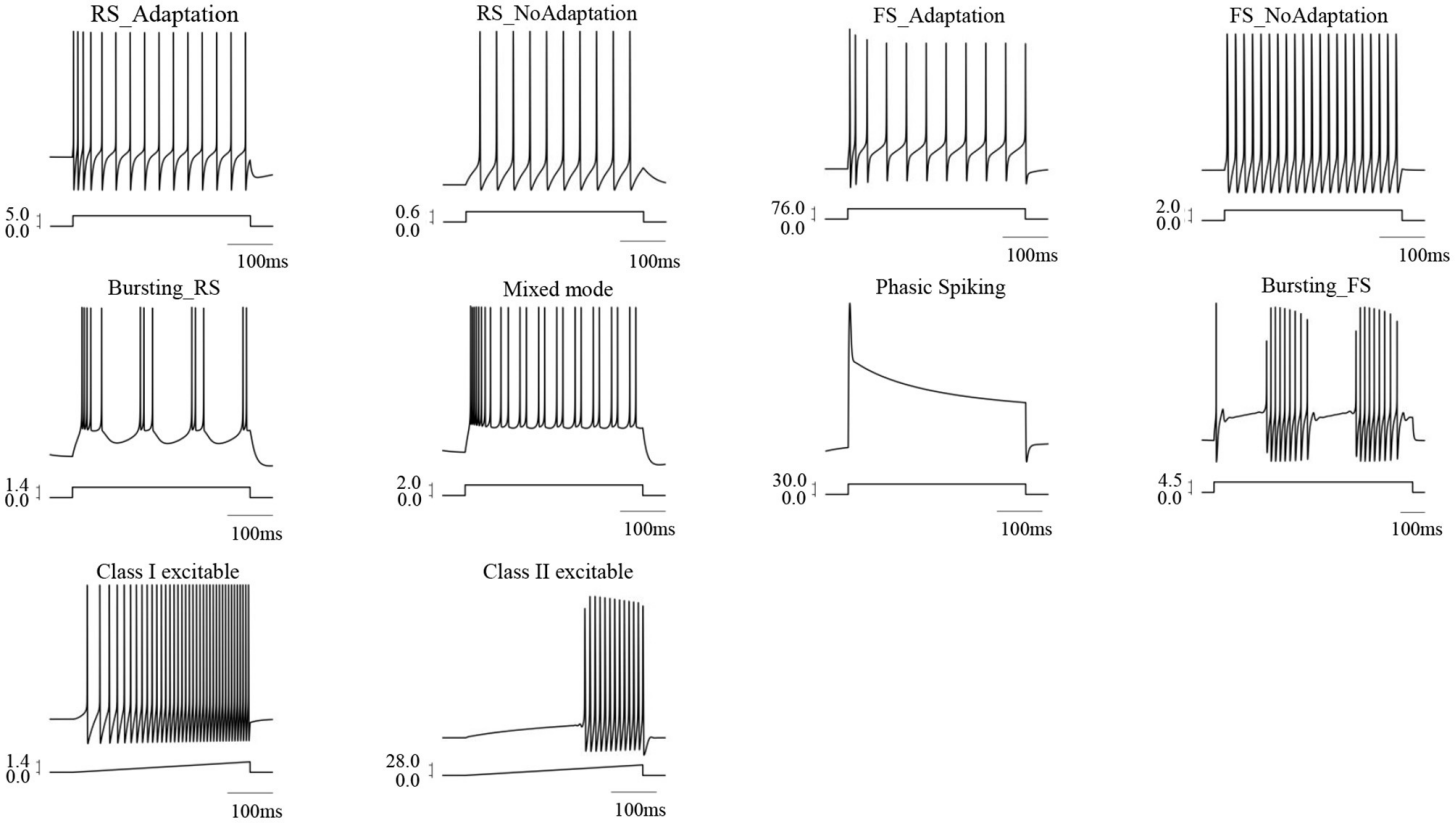
Ten typical firing patterns reproduced by nine different ionic models.

Spike data (*V*_*max*_, *V*_*min*_, *T*_*width*_, *V*_1_, *V*_2_, and *V*_3_) were collected from nine different ionic neuron models: regular spiking neuron with and without adaptation (RS_Adaptation Ermentrout, 1998 and RS_NoAdaptation Fohlmeister and Miller, 1997), fast spiking neuron with and without adaptation (FS_Adaptation Gouwens et al., 2010 and FS_NoAdaptaton Wang and Buzsáki, 1996), bursting excitatory neuron (Bursting_RS Golomb et al., 2006), bursting inhibitory neuron (Bursting_FS Golomb et al., 2007), phasic spiking neuron (Rothman and Manis, 2003; Gai et al., 2009), Class I firing excitable (Traub and Miles, 1991) and Class II firing excitable (Hodgkin and Huxley, 1952) neurons. Besides, the mixed mode can also be generated by Bursting_RS under certain stimulus intensity (Figure 3). These ten firing patterns cover most of the firing behaviors observed experimentally in different brain regions.

Detailed mathematical expressions for each neuron were described using the classical HH equation:


(1) $$\begin{array}{r} C_{m}\frac{dV}{dt}=-I_{leak}+I_{inj}-\underset{ion}{\sum }I_{ion} \end{array}$$

where *C*_*m*_ is the membrane capacitance, *I*_*leak*_ is the leak current, *I*_*inj*_ is the external input current with four modes: constant, slope, sinusoidal, and noise. $\underset{ion}{\sum }I_{ion}$ is the summation of ionic currents. *I*_*ion*_ is illustrated in equation 2.


(2) $$\begin{array}{r} I_{\text{ion}}=g_{\max}m^{p}n^{q}\left(V-E_{\text{ion}}\right) \end{array}$$

*g*_*max*_ is the maximal conductance of the corresponding ion channel, *m* and *n* are gating variables controlling the open and close states of ion channels, while *p* and *q* are the numbers of gating variables needed. *E*_*ion*_ is the reversal potential of each ion channel. Detailed expressions of these ion currents and electrophysiological parameters of RS_Adaptation, RS_NoAdaptation, FS_Adaptation, FS_NoAdaptation, Bursting_RS, Bursting_FS, Phasic spiking, Class I excitable, and Class II excitable were separately referred from Hodgkin and Huxley (1952), Traub and Miles (1991), Wang and Buzsáki (1996), Ermentrout (1998), Fohlmeister and Miller (1997), Golomb et al. (2006), Golomb et al. (2007), Rothman and Manis (2003), Gai et al. (2009), and Gouwens et al. (2010).

### 2.4. Collection of Spike Features and Voltage Samples for Training and Testing

For each specific model in Section 2.3, a corresponding dataset was built to train and evaluate the FPM. In the training stage, constant current with different intensities was used as the external input current to generate spike trains with different firing frequencies. For every spike, *V*_*max*_, *V*_*min*_, and *T*_*width*_, and sequential *V*_1_, *V*_2_, and *V*_3_ were collected in the data set. Here, the time interval between *V*_1_, *V*_2_, *V*_3_, and *V*_*max*_ is 1 ms. To test the generalization ability of the proposed model, three additional current stimuli were introduced during testing. Specifically, constant, slope, sinusoidal, and Gaussian white noise stimuli were used to evaluate the performance of FPM. The noise current mode was set as four levels, denoted as 1, 2, 3, and 4, by controlling the sigma of Gaussian distribution to make the noise current range cover 25, 50, 75, and 100% current range used in constant stimuli. The sample size of the training and testing data set for each neuron model is listed in Table 1. It should be mentioned that all the samples have labels.

**Table 1:** The sample size of the feature prediction module (FPM) training and testing data set used for each neuron model.

**Training**	**Testing**			
**Constant** **current**	**Constant** **current**	**Slope** **current**	**Noise** **current**	**Sinusoidal** **current**
2,400	2,400	2,400	9,600	2,400

In this study, voltage data of the nine neuron models were calculated in Python using the fourth-order Runge-Kutta algorithm with time integration of 0.01 ms. Construction of the FPM was realized in a Python-based package-PyBrain.

## 3. Results

### 3.1. Performance of the FPM in Training and Testing

**Figure 4:**
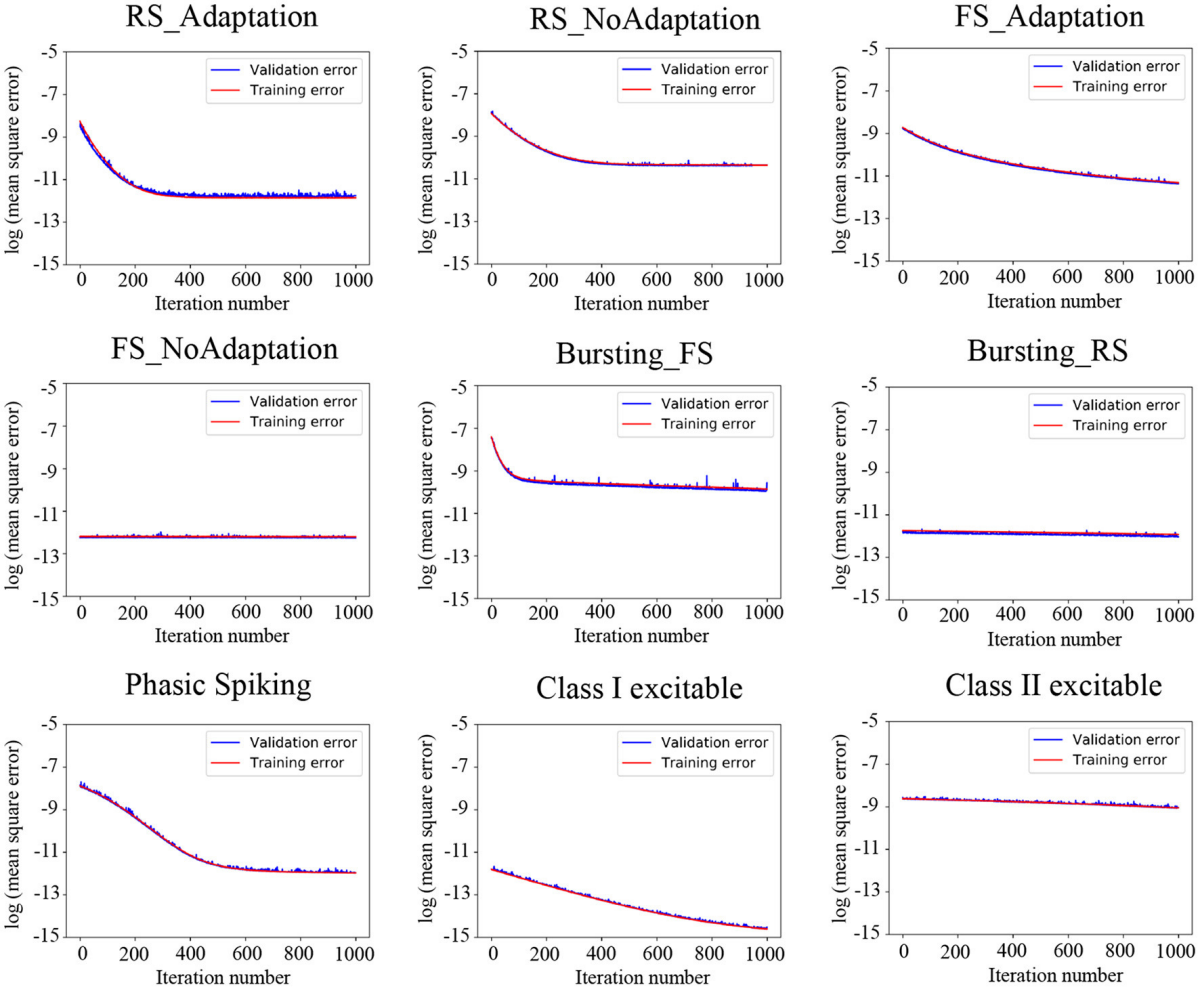
Training and validation error of FPM to the iteration number of nine models.

Voltage samples were regulated to the same distribution range for training and testing the FPM. Seventy percent of data in the training set were randomly selected for training, and the remaining 30% was used for validation. After iteration for 1,000 times, the FPM has been trained well enough. Figure 4 illustrates the change of MSE during training.

To examine the performance of FPM in testing data, we calculated prediction errors and accuracy of all the models under constant, slope, sinusoidal, and noise stimuli, where the noise stimuli were set as four levels. Here, prediction error was calculated using root mean square error (RMSE), while accuracy was calculated using the coefficient of determination (*R*^2^) from the regression analysis. As shown in Table 2, for the three spike features (*V*_*max*_, *V*_*min*_, and *T*_*width*_), the proposed method shows low prediction errors and high prediction accuracy on nine different neurons whose firing patterns cover most of the firing behaviors observed experimentally in the brain. Also, it is worth noting that the proposed method achieved good performance on the three current types that had not been used in training.

**Table 2:** Prediction errors and accuracy using samples from the library in the FPM.

**Models**	**Current type**		**RMSE (*V*_*max*_)**	**RMSE (*V*_*min*_)**	**RMSE (*T*_*width*_)**	***R*^2^ (*V*_*max*_)**	***R*^2^ (*V*_*min*_)**	***R*^2^ (*Ts*_*width*_)**
							
RS_Adaptation	constant		4.63 × 10^−3^	3.17 × 10^−3^	2.38 × 10^−3^	1.00	1.00	1.00
	slope		4.91 × 10^−3^	1.76 × 10^−2^	1.70 × 10^−3^	1.00	1.00	1.00
	sinusoidal		3.66 × 10^−3^	5.02 × 10^−4^	2.38 × 10^−3^	1.00	1.00	1.00
	noise	level=1	5.98 × 10^−4^	4.84 × 10^−3^	1.72 × 10^−3^	1.00	1.00	1.00
		level=2	8.17 × 10^−4^	6.57 × 10^−3^	1.65 × 10^−3^	1.00	1.00	1.00
		level=3	3.26 × 10^−4^	8.98 × 10^−3^	1.61 × 10^−3^	1.00	1.00	1.00
		level=4	1.67 × 10^−3^	5.84 × 10^−3^	1.78 × 10^−3^	1.00	1.00	1.00
RS_NoAdaptation	constant		1.26 × 10^−2^	1.34 × 10^−2^	8.85 × 10^−3^	1.00	1.00	1.00
	slope		1.20 × 10^−2^	1.30 × 10^−2^	8.85 × 10^−3^	1.00	1.00	1.00
	sinusoidal		1.94 × 10^−2^	2.86 × 10^−2^	9.90 ×10^−3^	1.00	1.00	1.00
	noise	level=1	3.23 × 10^−3^	7.33 × 10^−3^	3.48 × 10^−3^	1.00	1.00	1.00
		level=2	1.15 × 10^−3^	4.70 × 10^−3^	4.99 × 10^−4^	1.00	1.00	1.00
		level=3	3.86 × 10^−3^	4.24 × 10^−3^	5.52 × 10^−4^	1.00	1.00	1.00
		level=4	8.04 × 10^−3^	5.81 × 10^−3^	6.95 × 10^−4^	1.00	1.00	1.00
FS_Adaptation	constant		4.78 × 10^−1^	1.52 × 10^−1^	9.34 × 10^−3^	1.00	1.00	1.00
	slope		4.78 × 10^−1^	1.52 × 10^−1^	9.35 × 10^−3^	1.00	1.00	1.00
	sinusoidal		4.80 × 10^−1^	1.55 × 10^−1^	9.43 × 10^−3^	1.00	1.00	1.00
	noise	level=1	4.65 × 10^−1^	2.16 × 10^−1^	5.90 × 10^−3^	1.00	1.00	1.00
		level=2	4.61 × 10^−1^	2.16 × 10^−1^	5.89 × 10^−3^	1.00	1.00	1.00
		level=3	4.58 × 10^−1^	2.15 × 10^−1^	5.88 × 10^−3^	1.00	1.00	1.00
		level=4	4.37 × 10^−1^	2.05 × 10^−1^	5.44 × 10^−3^	1.00	1.00	1.00
FS_NoAdaptation	constant		1.93 × 10^−1^	1.91 × 10^−1^	2.65 × 10^−2^	1.00	1.00	1.00
	slope		1.93 × 10^−1^	1.90 × 10^−1^	2.64 × 10^−2^	1.00	1.00	1.00
	sinusoidal		1.86 × 10^−1^	1.78 × 10^−1^	2.22 × 10^−2^	1.00	1.00	1.00
	noise	level=1	7.01 × 10^−2^	6.50 × 10^−2^	4.64 × 10^−3^	1.00	1.00	1.00
		level=2	16.80 × 10^−2^	7.56 × 10^−2^	4.67 × 10^−3^	1.00	1.00	1.00
		level=3	7.70 × 10^−2^	9.90 × 10^−2^	8.92 × 10^−3^	1.00	1.00	1.00
		level=4	8.56 × 10^−2^	1.11 × 10^−1^	4.97 × 10^−3^	1.00	1.00	1.00
Bursting_RS	constant		1.18 × 10^−2^	6.77 × 10^−3^	1.34 × 10^−3^	1.00	1.00	1.00
	slope		1.10 × 10^−2^	6.20 × 10^−3^	1.33 × 10^−3^	1.00	1.00	1.00
	sinusoidal		3.87 × 10^−4^	6.29 × 10^−3^	2.33 × 10^−4^	1.00	1.00	1.00
	noise	level=1	1.00 × 10^−3^	8.85 × 10^−3^	9.01 × 10^−3^	1.00	1.00	1.00
		level=2	1.48 × 10^−3^	1.49 × 10^−2^	3.75 × 10^−2^	1.00	1.00	1.00
		level=3	1.38 × 10^−3^	1.28 × 10^−2^	8.89 × 10^−3^	1.00	1.00	1.00
		level=4	1.57 × 10^−3^	2.69 × 10^−2^	3.34 × 10^−2^	1.00	1.00	1.00
Bursting_FS	constant		2.67 × 10^−2^	2.27 × 10^−2^	9.34 × 10^−4^	1.00	1.00	1.00
	slope		2.58 × 10^−2^	2.35 × 10^−2^	9.08 × 10^−4^	1.00	1.00	1.00
	sinusoidal		2.56 × 10^−2^	1.12 × 10^−2^	7.89 × 10^−4^	1.00	1.00	1.00
	noise	level=1	3.13 × 10^−2^	2.41 × 10^−2^	9.21 × 10^−4^	1.00	1.00	1.00
		level=2	3.35 × 10^−2^	2.60 × 10^−2^	7.50 × 10^−4^	1.00	1.00	1.00
		level=3	2.36 × 10^−2^	3.27 × 10^−2^	4.77 × 10^−4^	1.00	1.00	1.00
		level=4	1.19 × 10^−2^	4.14 × 10^−2^	5.86 × 10^−4^	1.00	1.00	1.00
Phasic_Spiking	constant		6.26 × 10^−2^	8.23 × 10^−4^	2.67 × 10^−3^	1.00	1.00	1.00
	slope		6.24 × 10^−2^	7.81 × 10^−4^	2.67 × 10^−3^	1.00	1.00	1.00
	sinusoidal		1.06 × 10^−1^	3.02 × 10^−3^	2.55 × 10^−3^	1.00	1.00	1.00
	noise	level=1	4.69 × 10^−3^	7.50 × 10^−4^	3.32 × 10^−3^	1.00	1.00	1.00
		level=2	1.70 × 10^−2^	2.33 × 10^−4^	3.30 × 10^−3^	1.00	1.00	1.00
		level=3	2.81 × 10^−2^	7.49 × 10^−4^	3.29 × 10^−3^	1.00	1.00	1.00
		level=4	3.92 × 10^−2^	3.16 × 10^−3^	7.97 × 10^−4^	1.00	1.00	1.00
Class I excitable	constant		8.48 × 10^−4^	4.10 × 10^−3^	3.94 × 10^−4^	1.00	1.00	1.00
	slope		6.91 × 10^−4^	3.95 × 10^−3^	4.05 × 10^−4^	1.00	1.00	1.00
	sinusoidal		5.40 × 10^−3^	2.37 × 10^−3^	1.09 × 10^−4^	1.00	1.00	1.00
	noise	level=1	7.95 × 10^−4^	5.89 × 10^−3^	1.94 × 10^−3^	1.00	1.00	1.00
		level=2	9.04 × 10^−4^	4.75 × 10^−3^	1.72 × 10^−3^	1.00	1.00	1.00
		level=3	9.09 × 10^−5^	3.63 × 10^−3^	1.69 × 10^−3^	1.00	1.00	1.00
		level=4	7.17 × 10^−4^	3.97 × 10^−3^	1.78 × 10^−3^	1.00	1.00	1.00
Class II excitable	constant		1.31 × 10^−1^	4.10 × 10^−2^	1.38 × 10^−3^	1.00	1.00	1.00
	slope		1.31 × 10^−1^	4.10 × 10^−2^	1.38 × 10^−3^	1.00	1.00	1.00
	sinusoidal		1.59 × 10^−2^	1.70 × 10^−3^	1.52 × 10^−3^	1.00	1.00	1.00
	noise	level=1	3.63 × 10^−2^	9.36 × 10^−3^	1.37 × 10^−3^	1.00	1.00	1.00
		level=2	2.95 × 10^−2^	8.55 × 10^−3^	1.36 × 10^−3^	1.00	1.00	1.00
		level=3	1.62 × 10^−2^	3.47 × 10^−4^	5.42 × 10^−3^	1.00	1.00	1.00
		level=4	1.34 × 10^−3^	2.82 × 10^−3^	5.36 × 10^−3^	1.00	1.00	1.00

These results show that for a given sequential *V*_1_, *V*_2_, and *V*_3_, the trained FPM performs very well in predicting the features of the subsequent spike and demonstrates the robustness of the proposed model.

### 3.2. Visualization of the Performance of SPM and FPM in Spike Prediction

**Figure 5:**
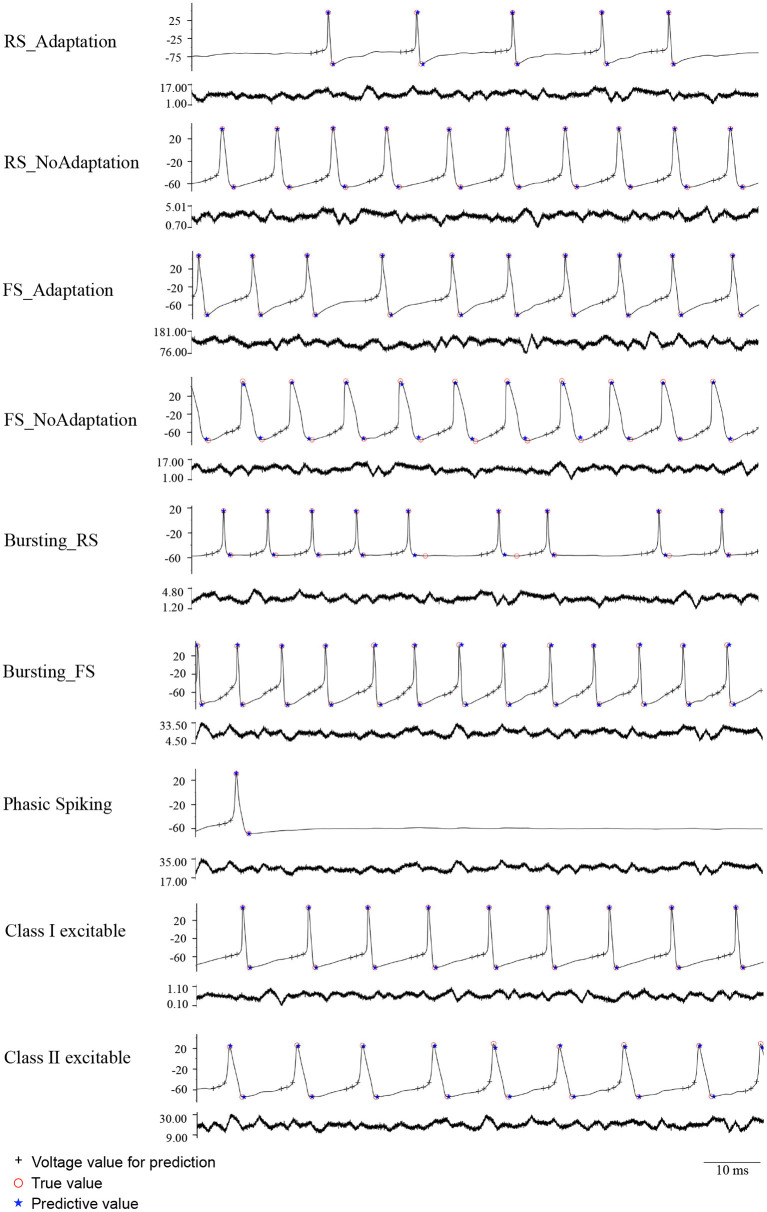
Nine neuron models showing the performance of SPM and FPM in prediction under noise current stimuli mode (level=4). Black crosses denote voltage values for spike prediction, red circles denote true values, and blue stars denote predictive values.

To intuitively demonstrate the performance of the proposed method, we visualized its prediction results under different external input currents. Figure 5 demonstrates representative neuron activities showing the performance of SPM and FPM in classification and prediction under noise current mode (noise level=4), from which we can see that the predictive values (blue stars) almost coincide with the true values (red circles). The predicted spike features under constant, slope, sinusoidal, and noise (level=1, 2, 3) modes are presented in the Supplementary Material.

## 4. Discussion

### 4.1. Contributions of This Study

Neuron models used in spiking neural networks can be divided into simplified models without ion channels, represented by the LIF model, and detailed models with ion channels, represented by the HH-type model. LIF models are computationally efficient and have been used in simulating large-scale brain networks and scenarios like dealing with asynchronous event-based spatio-temporal information (Yang et al., 2021a). However, LIF models might be inaccurate when obtaining spike features as they fail to capture detailed ion channel information. While HH-type models can simulate spike features more accurately, they suffer from computational complexity. In this study, we focus on HH-type neurons and built an artificial neural network, FPM, to provide novel insights of predicting subsequent spike features (*V*_*max*_, *V*_*min*_, and *T*_*width*_). While the previously proposed SPM can predict a spike event, the FPM can successfully predict informative spike features (*V*_*max*_, *V*_*min*_, and *T*_*width*_) for ten firing patterns that cover most of the firing behaviors observed in the brain. Also, it can be well generalized to different unseen input current scenarios. Experimental results demonstrate that the combination of SPM and FPM can successfully predict the spiking part of nine different HH-type neuron models, offering a possible efficient way of simulating action potentials of biological neurons with high accuracy.

### 4.2. Diverse Firing Patterns in Neurons

Firing patterns exhibited by neurons reflect what coding strategies or manners these neurons adapt to encode input signals. During the past decades, a diversity of firing patterns has been observed in biophysical experiments (summarized in Izhikevich, 2004), e.g., spiking, bursting, and oscillation. Besides, spiking can be further classified into regular spiking (with and without adaptation), fast spiking (with and without adaptation), and phasic spiking, etc., while bursting can be further classified into regular bursting, fast bursting, phasic bursting, and rebound bursting, and so on. Based on the properties of frequency response (f) to different injected current (I), f-I curves and dynamical firing structures, firing behaviors of neurons can be classified into Class I, Class II, and Class III types. In this study, to test the effectiveness of our proposed method, nine ionic models which can generate most of the firing patterns mentioned above have been considered, including RS_Adaptation, RS_NoAdaptation, FS_Adaptation, FS_NoAdaptation, phasic spiking, Bursting_RS, Bursting_FS, mixed-mode, and class I and class II excitable neurons. The obtained results not only demonstrate the reliability of our method in predicting spike features but also show the generality of our method in applying to diverse neuron models or firing patterns.

### 4.3. Other Methods in Solving HH Equations

For a long period, numerical methods used to solve differential equations or models were Euler, Runge- Kutta, and some other revised versions. To achieve higher accuracy in solving equations, relatively smaller time steps were usually employed, e.g., 0.01 and 0.005 ms. However, the reduction of time steps would sacrifice the computation speed. For models with few equations, computational speed may not be a problem, while for models with plenty of equations, computation speed will be very critical.

This problem is especially serious in simulating large-scale neural network models. To address this problem, a novel library-based method has been proposed to accelerate the speed of action potential calculations (Sun et al., 2009). However, it can only acquire raw statistical information of spikes and is not able to capture the relative spike timing information. Also, its generalization ability is unknown as the method has only been tested in the classical HH model.

In this study, our proposed method has been tested in various ionic neuron models. Experimental results demonstrate that the proposed method not only performs well in accurately simulating action potentials of neurons but also shows good generalities to different HH-type neuron models and firing patterns.

### 4.4. Extensions of the Current Study

Our proposed method performed well in predicting spike features in many neuronal models. We speculate that the combination of SPM and FPM may contribute to an overall acceleration in the adoption of the HH equation. Specifically, the SPM and FPM can be used to effectively predict the spiking parts during fast voltage-changing periods, when the voltage is relatively stable and changes slowly, a larger time step can be implemented to speed up the computation.

While a previous study has tried to approximate the characteristics of a multi-compartment neuron by a temporally convolutional neural network with five to eight layers (Beniaguev et al., 2021), for situations that can be handled with point neurons, our proposed method can be effectively applied to predict the spiking part of each neuron. Also, the proposed method may be extended to large-scale network simulation by replacing traditional HH-type neurons with simple ANNs.

Although our proposed method performed well in predicting spike features in many neuronal models, there are still some insufficiencies that need to be improved in further studies. In this study, all neuron models considered are point neuron models. It is unknown if the method can be extended to more complicated situations like CerebelluMorphic (Yang et al., 2021c) and large-scale biophysically meaningful neural networks with multi-compartmental neurons (Yang et al., 2021b). Further studies are needed.

## Data Availability Statement

The code and dataset presented in the study are publicly available. This data can be found here: https://github.com/tianaeiou/Spike-Prediction.

## Author Contributions

LC designed the study. YW, TW, JS, and LW proposed the analysis. YW, TW, LW, and LC wrote the paper. All authors contributed to the article and approved the submitted version.

## Funding

This work was supported by the National Natural Science Foundation of China (grant no. 62176241), the National Key Research and Development Program of China (grant no. 2021ZD0200300), and the Open Project Program of the State Key Laboratory of Mathematical Engineering and Advanced Computing (grant no. 2020A09).

## Conflict of Interest

The authors declare that the research was conducted in the absence of any commercial or financial relationships that could be construed as a potential conflict of interest.

## Publisher's Note

All claims expressed in this article are solely those of the authors and do not necessarily represent those of their affiliated organizations, or those of the publisher, the editors and the reviewers. Any product that may be evaluated in this article, or claim that may be made by its manufacturer, is not guaranteed or endorsed by the publisher.
