# Supplementary material for: Predicting Spike Features of Hodgkin-Huxley-Type Neurons With Simple Artificial Neural Network
Source: Front Comput Neurosci. 2022 Feb 7;15:800875. doi: 10.3389/fncom.2021.800875 (PMC8859780; doi:10.3389/fncom.2021.800875)
Supplement: Supplementary file 1 [file Data_Sheet_1.PDF]

# *Predicting Spike Features of Hodgkin-Huxley-type Neurons with Simple Artificial Neural Network*

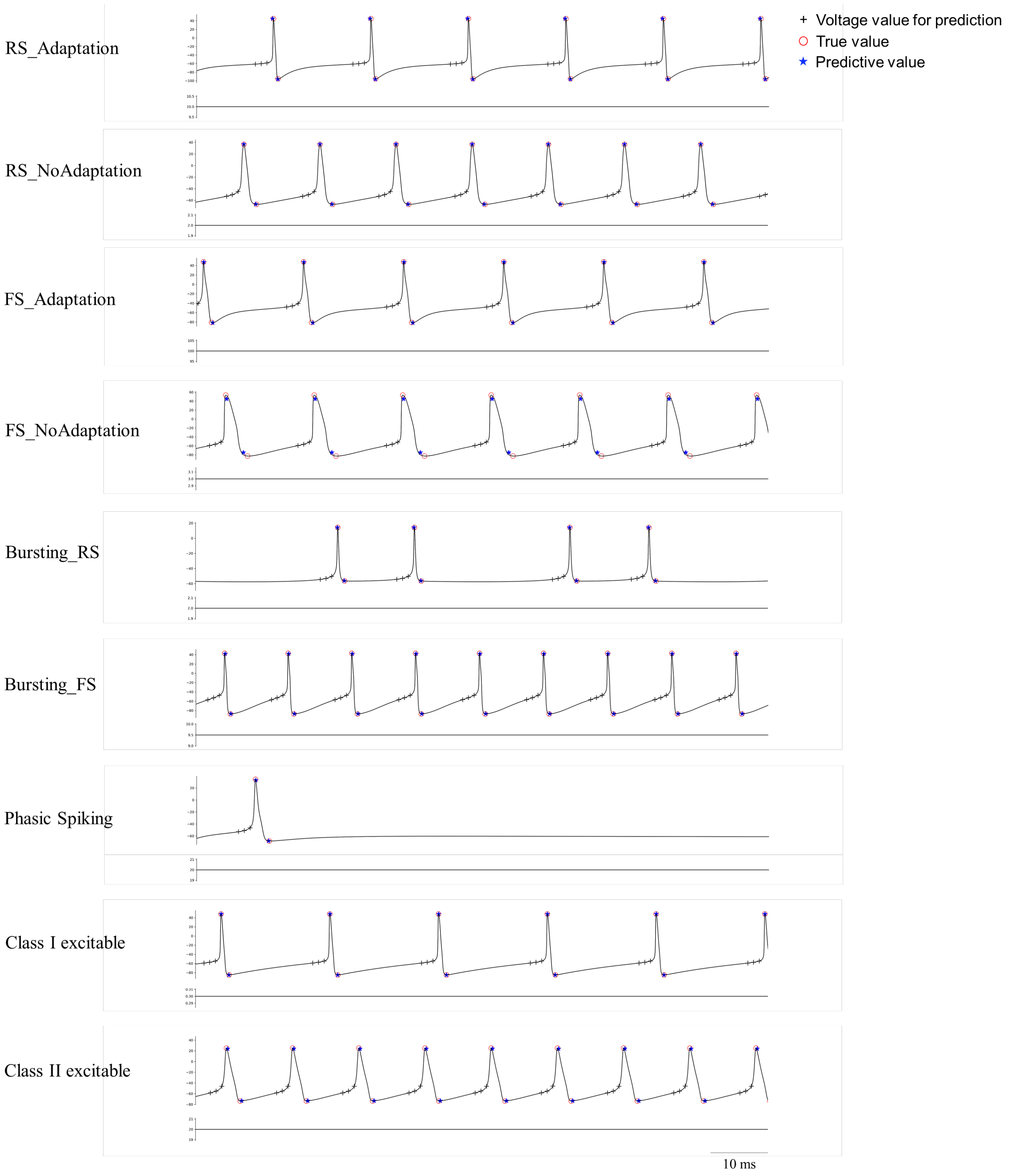


**Figure S1.** Nine neuron models showing the performance of SPM and FPM in prediction under constant current stimuli mode. Black crosses denote voltage values for spike prediction, red circles denote true values, while blue stars denote predictive values.

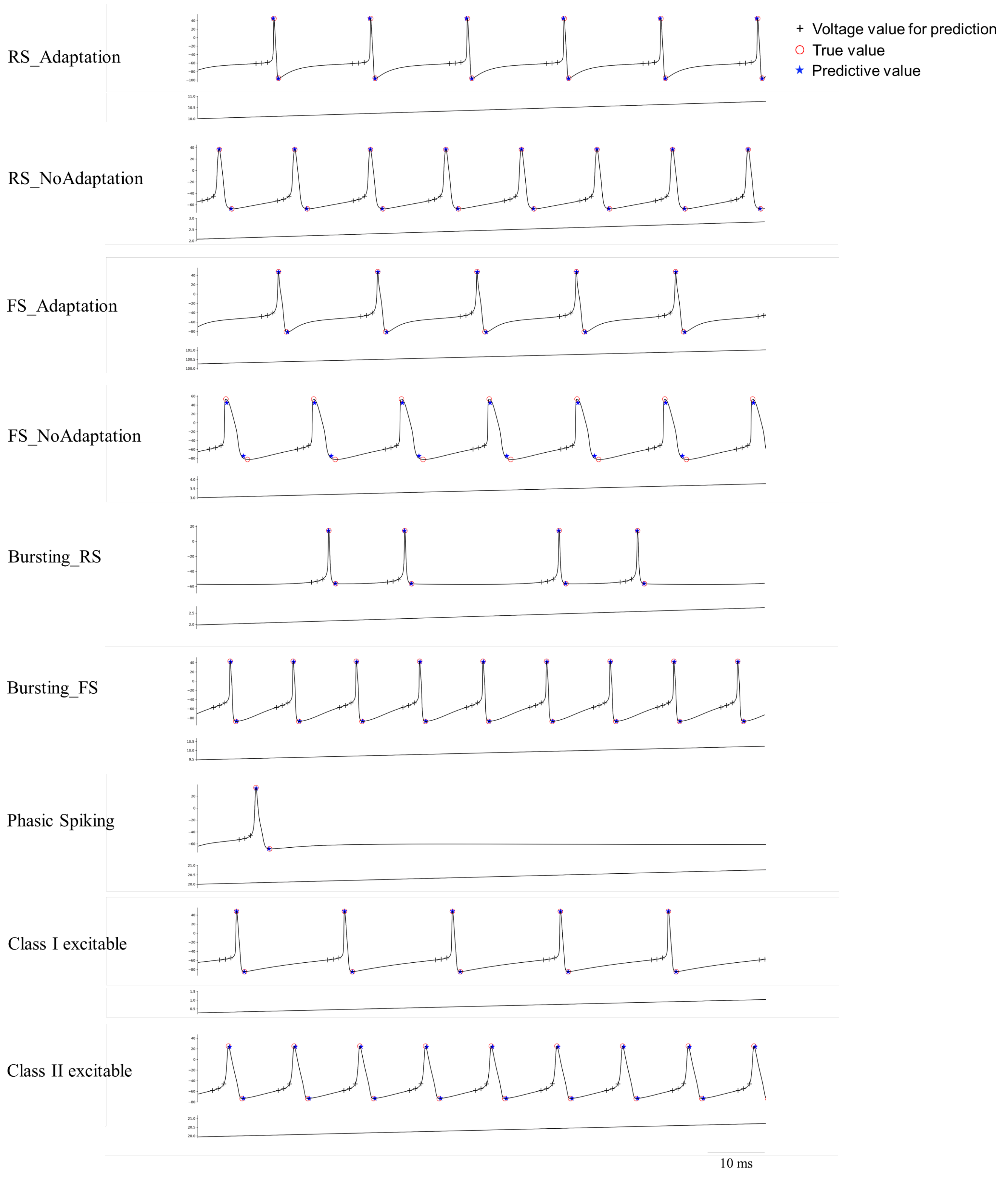


**Figure S2.** Nine neuron models showing the performance of SPM and FPM in prediction under slope current stimuli mode. Black crosses denote voltage values for spike prediction, red circles denote true values, while blue stars denote predictive values.

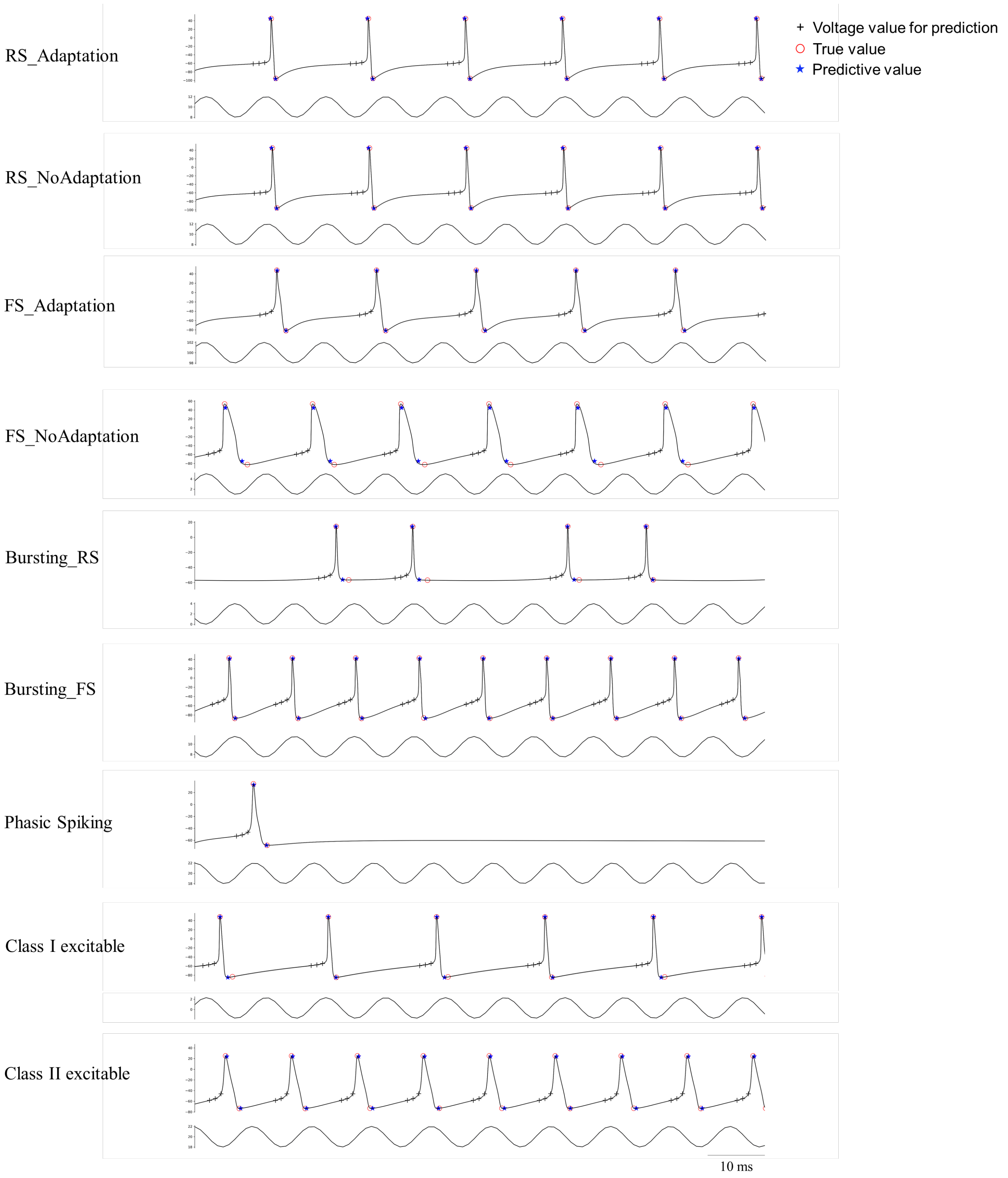


**Figure S3.** Nine neuron models showing the performance of SPM and FPM in prediction under sinusoidal current stimuli mode. Black crosses denote voltage values for spike prediction, red circles denote true values, while blue stars denote predictive values.

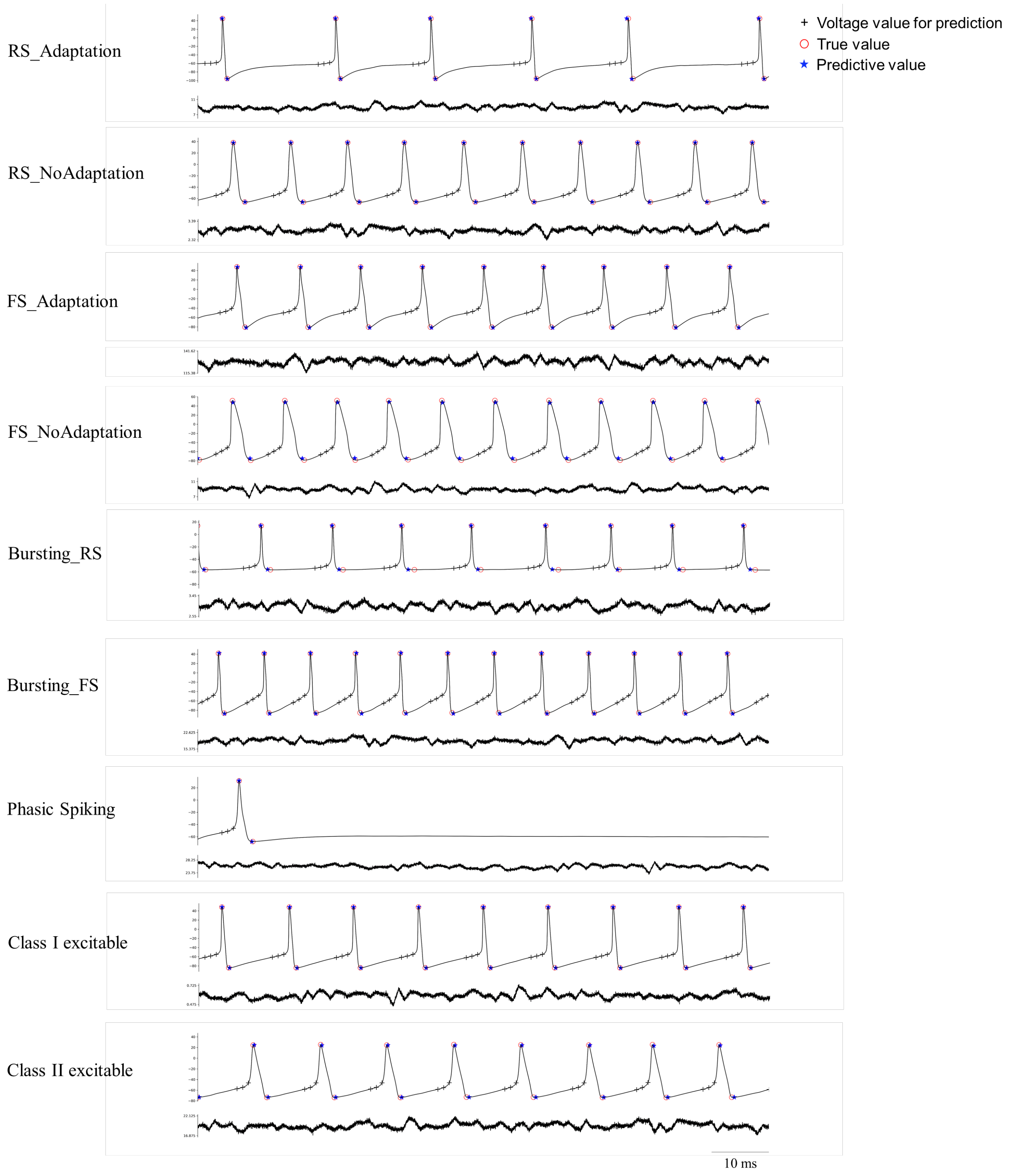


**Figure S4.** Nine neuron models showing the performance of SPM and FPM in prediction under noise current stimuli mode (level=1). Black crosses denote voltage values for spike prediction, red circles denote true values, while blue stars denote predictive values.

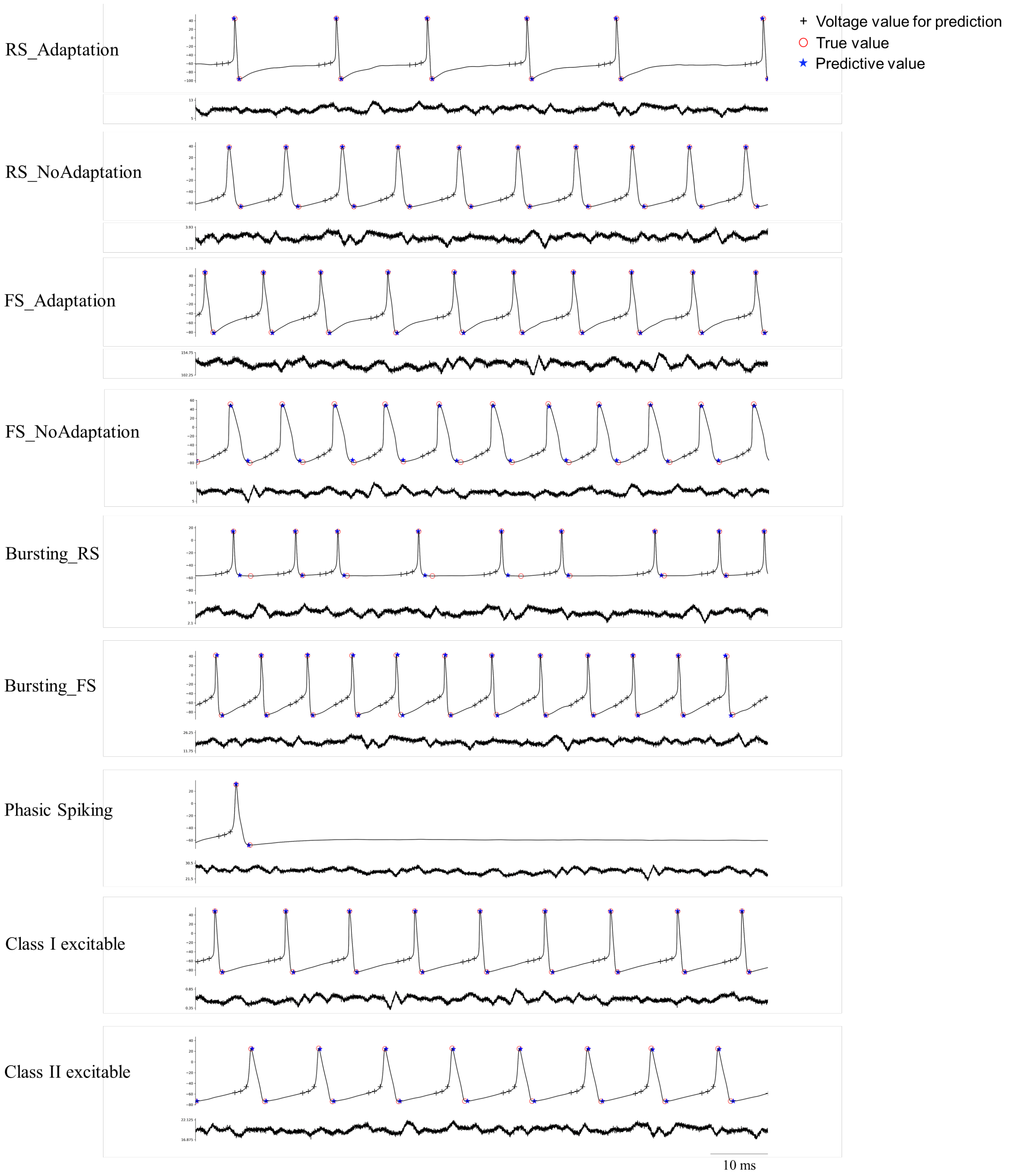


**Figure S5.** Nine neuron models showing the performance of SPM and FPM in prediction under noise current stimuli mode (level=2). Black crosses denote voltage values for spike prediction, red circles denote true values, while blue stars denote predictive values.

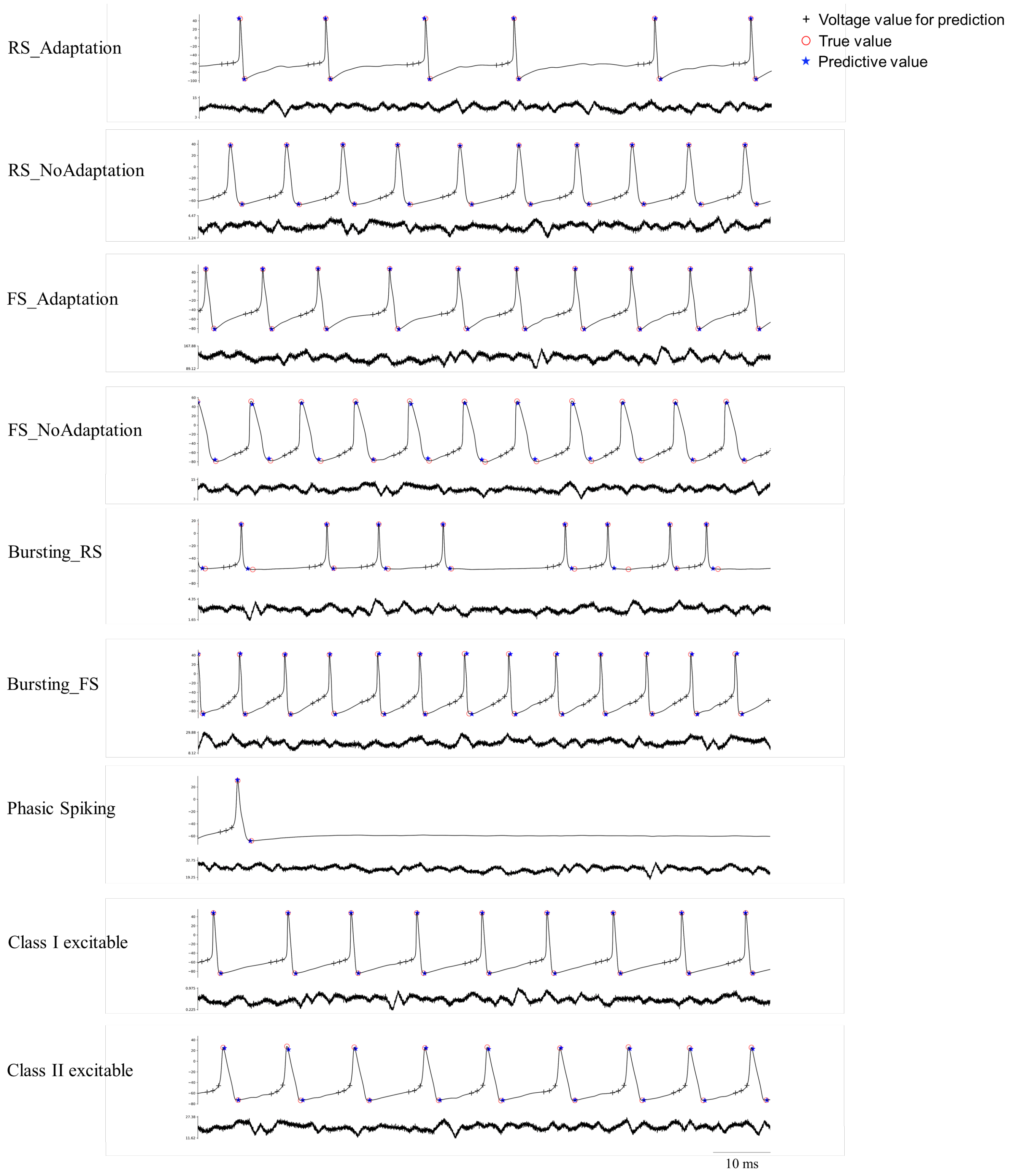


**Figure S6.** Nine neuron models showing the performance of SPM and FPM in prediction under noise current stimuli mode (level=3). Black crosses denote voltage values for spike prediction, red circles denote true values, while blue stars denote predictive values.
